# Health Characteristics of Solo Grandparent Caregivers and Single Parents: A Comparative Profile Using the Behavior Risk Factor Surveillance Survey

**DOI:** 10.1155/2015/630717

**Published:** 2015-09-10

**Authors:** Deborah M. Whitley, Esme Fuller-Thomson, Sarah Brennenstuhl

**Affiliations:** ^1^School of Social Work, Andrew Young School of Policy Studies, Georgia State University, P.O. Box 3993, Atlanta, GA 30302, USA; ^2^Factor-Inwentash Faculty of Social Work, University of Toronto, 246 Bloor Street West, Toronto, ON, Canada M5S 1A1; ^3^Institute for Life Course & Aging, University of Toronto, 263 McCaul Street, Suite 328, Toronto, ON, Canada M5T 1W7

## Abstract

*Objectives*. To describe the health characteristics of solo grandparents raising grandchildren compared with single parents. *Methods*. Using the 2012 Behavioral Risk Factor Surveillance System, respondents identified as a single grandparent raising a grandchild were categorized as a *solo grandparent*; grandparent responses were compared with single parents. Descriptive analysis compared health characteristics of 925 solo grandparents with 7,786 single parents. *Results*. Compared to single parents, grandparents have a higher prevalence of physical health problems (e.g., arthritis). Both parent groups have a high prevalence of lifetime depression. A larger share of grandparents actively smoke and did no recreational physical exercise in the last month. However, grandparents appear to have better access to health services in comparison with single parents. *Conclusion*. Solo grandparents may be at risk for diminished physical capacity and heightened prevalence of depression. Health professionals can be an important resource to increase grandparents' physical and emotional capacities.

## 1. Introduction

It has become standard practice in child welfare agencies to place abused and neglected children removed from parental custody into the care of a family member, often a grandparent. The U.S. Census Bureau estimates there were 2.7 million grandparent caregivers in 2012, the majority being grandmothers [1]. These grandparents had total custodial care of their grandchildren because the parents had no active or consistent participation in rearing them. However, undertaking a long-term parental role takes its physical and emotional toll on grandparents, especially when performing their responsibilities without the support of a spouse or significant other. For the purposes of this study, we term these caregivers “solo grandparents.” Despite the growing prevalence of grandparents raising grandchildren, knowledge of their physical and mental health status remains limited because earlier studies have tended to aggregate all custodial grandparents into a single study unit regardless of family composition. Previous works have also used various data collection methods, sample sizes, and different definitions of health. These variances hamper our ability to develop an accurate profile of the unique health characteristics of grandparent caregivers by subgroups, which would advance family-based research, intervention practices, and policy efforts. The next generation of research on custodial grandparents should disaggregate the population and discern unique subgroup characteristics, which is the focus of the current study.

We selected single parents raising children as a comparison group because they appear to share similar characteristics with solo grandparents. In 2012, approximately 14% of all family groups with children were headed by single parents (mother only and father only households) [2]. Census data suggests many of these families were headed by mothers, who were never married, were economically disadvantaged, resided in the southern region of the country, and received some form of public assistance. Similarly, of the 2.7 million grandparent caregivers, 39% have been caring for their grandchildren 5 or more years, 45% live in the south and southwest regions, and approximately 17% live below the poverty level [1]. The magnitude of these numbers becomes apparent when the number of grandchildren being raised in these households is realized. In 2013, 2.9 million grandchildren were under the care of their grandparents, and 33% of these children are in parent-absent households [3]. Clearly, a significant segment of the future generation of adults is impacted by the care received as children by their grandparents. If the grandparents had not stepped forward to provide care, many of these children would be in the public foster care system where the potential risks for poor developmental, social, and emotional outcomes are well documented [4]. Therefore, the objective of the present study is to describe and compare five self-reported health characteristics (physical health, mental health, functional capacity, health behaviors, and health care utilization practices) using a representative sample from 36 U.S. states.

## 2. Literature Review

The health of grandparent caregivers has received considerable attention in the literature, especially in recent years. Several authors have documented grandparent caregivers' self-reports of physical health [5–10]. Higher levels of obesity, hypertension, heart disease, limited physical functioning, and dissatisfaction with one's physical health are frequently mentioned in the literature [11–14]. Emotional stress from financial constraints, inadequate social supports, and raising multiple grandchildren with significant emotional, developmental, and physical disabilities are additional challenges many grandparents reportedly experience [15, 16]. Some health problems are related to the grandparents' natural aging processes (e.g., arthritis), while others are linked to emotional strains from traumatic life challenges and burdens. As a result, previous studies conclude custodial grandparents are at high risk for depression, low physical functioning, and poor self-perception of their health [17, 18]. While grandparents may have the ability to access medical services to support their health challenges through available health insurance programs (e.g., Medicare), some studies suggest they lack knowledge about the availability of specific or specialized services, experience time constraints due to parenting responsibilities, or have limited transportation to available health service sites [19, 20]. Therefore, generalizing previous findings on the health of custodial grandparents to the population has its challenges since studies have tended to use small, nonrepresentative samples, classified by gender (e.g., grandmothers) or race (e.g., African Americans), with little to no consideration of other categories of family characteristics [21].

Another body of research focuses on the effects of caregiving on the physical health of grandparents [13, 22–24]. There is evidence suggesting the stress of caregiving worsens grandparents' health status [21, 24]. Some grandparents delay or neglect their own health care needs in order to address the more pressing needs of their grandchildren [25]. Grandmothers new to the caregiving role are least likely to participate in health promotion activities, as compared to grandmothers who raised their grandchildren two or more years, or never had to raise their grandchildren [26]. A possible effect of any medical delay is the intensification of symptoms associated with existing chronic illnesses, potentially leading to rapid physical decline. Certainly, a grandparent's diminished health status might have a negative impact on the ability to carry out necessary parenting roles and responsibilities for grandchildren in care.

Another group of studies addresses the effectiveness of intervention services on the health of grandparent caregivers [12, 18, 27, 28]. Support groups, home visiting, case management, legal support, and respite care are examples of intervention services offered to custodial grandparents. Findings from these types of studies suggest direct support services to custodial grandparents can have a positive effect on their physical health. Grandparents participating in these programs are reported to have improved self-care health practices and better perceptions of their mental and physical health [18, 28].

### 2.1. Single Parent's Health

The health of single parents seems to parallel that of custodial grandparents. Single parents raising children may also experience poor physical and psychological well-being, with the greatest burden occurring among single mothers as opposed to single fathers [29–32]. Stress from parenting responsibilities, financial strains, and emotional difficulties stemming from family conflicts are known to have adverse effects on the psychological well-being of single mothers, increasing risks for psychological distress and depression [33, 34]. Researchers have found that when single parents are experiencing financial hardship and lack access to social supports, they also have an increased risk of poor physical and emotional outcomes [35], which is similar to custodial grandparents. Hypertension, obesity, risk of diabetes, and high cholesterol levels are often reported among single mothers, especially those who are economically disadvantaged [36]. Poor, single mothers also may be more likely to display health behaviors (e.g., current smoking and lack of physical activity) that increase risk for chronic illnesses [37].

## 3. Theoretical Framework

The present study draws on the Transactional Model of Stress and Coping (TSC). The origins of the model stem from the formative work of Lazarus and Folkman [38, 39]. According to the authors, a stressor is a life demand that can influence individuals' physical and mental health depending on how they appraise it with respect to whether they perceive it as a threat or as irrelevant (primary appraisal). If a stressor is perceived as a threat, an individual then determines if they have the coping mechanisms to mitigate it (secondary appraisal). Specific coping behaviors to ameliorate stress can have an impact on emotional well-being, functioning, and health behaviors. Ongoing stress is known to accelerate aging and physical health problems associated with morbidity and mortality [40]. Solo grandparents and single parents are vulnerable to adverse health effects if they are unable to cope with the stresses of child-rearing with the myriad of personal and familial challenges that accompanies the parental role.

With this framework in mind, and the literature on grandparenting and health, we expect that solo grandparents will fare significantly worse in each of five health domains (i.e., physical health, mental health, functional limitations, health behaviors, and health service utilization) than single parents. Conducting this type of study has merit because so little is known about solo grandparent health and the extent to which it is worse than single parents' health, who, although younger, deal with many of the same stressors. Potentially, this research will reveal new perspectives on health care for both family groups at risk for poor clinical outcomes and the role of health care professionals to assist them.

## 4. Materials and Methods

### 4.1. Data Source and Sample

The data for our study are derived from the 2012 Behavioral Risk Factor Surveillance System (BRFSS), a collaborative project of the Centers for Disease Control and Prevention (CDC) and the U.S. States and Territories. The BRFSS is an ongoing data collection program designed to assess state-specific behavioral risk factors for the adult population [41]. Each month, trained interviewers use a standardized questionnaire to collect data over the phone from a representative sample of noninstitutionalized adults aged 18 or older who are living in households. The BRFSS uses a disproportionate stratified sample design, which divides all possible phone numbers into high-density and medium-density groups that are sampled separately. The sample selected for the current study included 925 solo grandparents and 7,786 single parents, which are defined below. For our analyses, the sample was restricted to the 36 states that opted to include the random child selection module [42]. Questions from this module allowed us to identify if the caregiver was a grandparent. Although the BRFSS 2012 includes both cellular phones and landlines, the question on the number of adults in the household was only asked of those with landlines. We used this question to determine if the grandparent caregiver was solo. Thus, our findings are generalizable to community-dwelling individuals who have landlines in 36 states. The landline response rate for the BRFSS 2012 was 49.1% [41].

### 4.2. Measures

#### 4.2.1. Parental Identification

In the BRFSS, there is a module used in 36 states that focused on a randomly selected child under age of 18 in the household [42], hereafter referred to as the “focus child”. One of the questions asks about the adult respondent's relationship to the focus child. If the adult responded “grandparent” and there was only one adult in the household, this respondent was categorized as a “solo grandparent caregiver.” If the adult answered “parent,” which could include biologic, step, or adoptive, and there was only one adult in the household, this respondent was categorized as a “solo parent.”

#### 4.2.2. Child Characteristics

In the focus child module described above, there were questions on the focus child's age (<age 5, 5–12 years, and 13–18), gender, residency within a metropolitan statistical area (MSA) (inside center city, outside center city, inside suburban county, MSA with no center city, outside MSA), and ethnicity. Specific questions about health status concerned asthma (“Has a doctor, nurse, or other health professional ever said that the child has asthma?”) and obtaining a flu shot in the past year (“During the past 12 months, has (the child) had a seasonal flu vaccination?”). The number of children in the household was also recorded.

#### 4.2.3. Parental Demographics

Background information was gathered on the parent figure, including the following: gender (male versus female), age (18–39, 40s, 50s, 60s, 70s, and 80+), race (white non-Hispanic, black non-Hispanic, Hispanic, and other), education level (less than high school diploma versus greater), and income (<$15,000, $15,000–24,999, $25,000–49,999, $50,000–74,999, and $80,000 or greater).

#### 4.2.4. Physical and Mental Health Descriptions

BRFSS respondents were asked to indicate, from a listing of chronic conditions, whether a “doctor, nurse, or another health professional” ever told them they had any of the following: problems arthritis, coronary obstructive pulmonary disease (COPD), diabetes (excluding borderline and gestational), asthma, cancer (excluding skin), heart attack, stroke, angina or coronary heart disease, kidney disease, and depression. Self-assessed health was also measured using the following question: “Would you say that your health, in general, is excellent, very good, good, fair, poor?” Responses were recoded into excellent/very good/good health versus fair/poor health. Finally, health-related quality of life was measured based on a series of three questions. The first was “Now thinking about your physical health, which includes physical illness and injury, for how many days during the past 30 days was your physical health not good?” Responses were recoded into 0, 1–7, and >7 days. The same type of question was also asked about mental health (e.g., Now thinking about your mental health…); responses were recoded in a similar fashion as physical health. The third question was as follows: “During the past 30 days, for about how many days did poor physical or mental health keep you from doing your usual activities, such as self-care, work, or recreation?” Responses were recoded using the same categories as physical health (i.e., 0, 1–7, and >7 days).

#### 4.2.5. Health Care Utilization

Access to health care coverage was assessed by response to the following question: “Do you have any kind of health care coverage, including health insurance, prepaid plans such as HMOs, or government plans such as Medicare, or Indian Health Service?” Respondents were also asked about their access to doctors using the following two questions. The first question was, “Do you have one person you think of as your personal doctor or health care provider?” Responses were recoded into yes (one or more) and no. The second question was, “Was there a time in the past 12 months when you needed to see a doctor but could not because of cost?” Finally, the respondents were asked to give the time since their last routine check-up using the following question: “About how long has it been since you last visited a doctor for a routine check-up?” (A routine check-up is a general physical exam, not an exam for a specific injury, illness, or condition.) Response options were in the last year, 1-2 years, 2–5 five years, or more than five years.

#### 4.2.6. Health Behaviors

Specific health behaviors were assessed in the 2012 BRFSS [41]. The first question considered recreational physical exercise: “During the past month, other than your regular job, did you participate in any physical activities or exercise such as running, calisthenics, golf, gardening, or walking for exercise?” The second health behavior assessed was a Body Mass Index score, computed from three levels: neither overweight nor obese (BMI < 25), overweight (BMI ≥ 25 and <30), and obese (BMI ≥ 30). The third health behavior was computed smoking status categorized into four groups: current smoker, everyday; current smoker, some days; former smoker; and never smoked.

### 4.3. Data Analysis

We undertook a descriptive analysis to identify the demographic, physical, and mental health characteristics, functional limitations, health behaviors, and health services utilization of solo grandparents and contrasted them with single parents. Comparisons were made using chi-square tests undertaken in SPSS version 21. All percentages and *p* values were weighted to take into account the probability of selection and nonresponse bias.

## 5. Results

Beginning with demographic characteristics found in Table 1, we show that a slightly smaller proportion of solo grandparents is female (*p* < 0.05). As we expected, solo grandparents are significantly older in age as compared to single parents (*p* < 0.001).

The largest proportion of solo grandparents is in their 60s (32.5%), while almost half of single parents are between 18 and 34 years of age (48.3%). Differences by ethnic-racial groups are also apparent: a similar percentage of solo grandparents and parents self-identified as white, but a larger proportion of solo grandparents compared to single parents are African American, and a smaller percentage are Hispanic (*p* < 0.001). Solo grandparents have a lower socioeconomic status than single parents; almost a third of solo grandparents have not completed high school (31.2%) versus less than one-seventh of single parents (14.7%; *p* < 0.001). Just over a quarter of solo grandparents have incomes less than $15,000 per year (27.8%) compared to a fifth of single parents (20.2%; *p* < 0.001), and approximately one-fifth do not reside in a Metropolitan Statistical Area (21.4% versus 15.6% of single parents; *p* < 0.001). Turning to the demographics associated with the children, we find that a larger percentage of solo grandparents have only one child in the household than single parents (61.9% versus 42.8%; *p* < 0.001). There are no significant differences in the gender or age of children. Interestingly, a lower prevalence of children with asthma is found in homes of solo grandparents (15.8% versus 19.7; *p* < 0.05) as is a higher prevalence of children who had the flu shot in the last year (63.2% versus 50.3%; *p* < 0.001).

Table 2 provides the physical and mental health characteristics of our sample. As expected, a larger percentage of solo grandparents than parents report having conditions related to aging, including arthritis (50.5% versus 18.8%; *p* < 0.001), COPD (21.4% versus 4.2%; *p* < 0.001), diabetes (25.6% versus 6.1%; *p* < 0.001), cancer (13.2% versus 4.3%), heart attack (18.0% versus 1.4%; *p* < 0.001), stroke (8.7% versus 1.6%; *p* < 0.001), heart disease (11.2% versus 1.9%; *p* < 0.001), and kidney disease (6.9% versus 1.9%; *p* < 0.001). Solo grandparents also report having asthma at higher rates than single parents (22.9% versus 14.4%; *p* < 0.001) and have a slightly elevated prevalence of self-reported depression, but the difference did not reach statistical significance (27.3% versus 23.7%; *p* = 0.06). Consistent with information on physical health conditions, a higher percentage of solo grandparents report having fair or poor health (39.9% versus 16.6%; *p* < 0.001), more than seven days of bad physical health a month (31.7% versus 14.4%; *p* < 0.001), and/or limitations in daily activities (23.7% versus 11.8%; *p* < 0.001) than single parents. Finally, the health behaviors of solo grandparents are worse than those of single parents. A greater proportion of solo grandparents have not exercised recreationally in the last month (40.6% versus 24.2%; *p* < 0.001) and are current, everyday smokers (20.9% versus 15.8%) or former smokers (34.5% versus 17.8%; *p* < 0.001) than single parents. There are no significant differences, however, in the percentage of those who are obese by caregiver type.

Responses to health utilization characteristics are found in Table 3. More than 90% of the solo grandparents in our sample have health care coverage compared to just over four-fifths of single parents (91.6% versus 81.1%; *p* < 0.001).

A larger percentage of solo grandparents also report having a regular personal doctor (87.7% versus 81.6%; *p* < 0.001), while a smaller proportion report not seeing a doctor because of cost (18.6% versus 22.4%; *p* < 0.05). Finally, almost four-fifths of solo grandparents had a routine check-up in the last year, compared to just over two-thirds of single parents (*p* < 0.001).

## 6. Discussion

The present study describes the health characteristics of solo grandparents raising grandchildren in comparison with single parents, using a national dataset. While some studies have used smaller samples to describe the health of grandparent caregivers, our study attempts to determine if those results can be replicated in a large, representative sample. Also, other studies identify “skipped-generation” households, wherein the birth parents are not living at home, but there is usually no confirmation as to whether the grandparent caregiver was the* sole* caregiver in the household. The BRFSS, however, captures custodial grandparents across various ages and family arrangements and, thus, our results extend current knowledge in the field about the health status of grandparents raising grandchildren. Our general expectation that solo grandparents would fare worse than single parents was fully or partially confirmed across four health domains: physical health, mental health, functional limitation, and health behaviors.

### 6.1. Physical Health

The data show that solo grandparents have a higher prevalence of each of the physical health conditions measured. The high prevalence of health problems is consistent with the nearly 40% of grandparents who rated their physical health as “*fair*” or “*poor*,” and almost 32% who viewed their health as being “*not good*” for more than a week in a given month. The leading diagnoses among grandparents were arthritis, diabetes, asthma, and COPD. Single parents, as expected, were younger and healthier; their most prevalent diagnoses were arthritis and asthma, but at significantly reduced rates than grandparents. Age is likely a contributing factor for this finding because certain diagnoses are more prevalent in older adult population groups (e.g., arthritis, COPD). Our findings concur with earlier research that highlight the range of adverse physical health conditions that affect custodial grandparents. One of the implications of having a higher prevalence of physical health conditions is that solo grandparents typically need to spend more time addressing their own health concerns. Because solo grandparents are likely to have smaller social networks as compared to younger, single parents, access to other adults to share parenting responsibilities is limited. Offering solo grandparents access to support services such as daycare, respite, case management, and emergency childcare, thus, is likely vital [28, 43]. In addition, health education and health promotion interventions on diet, exercise, and medication adherence are equally important to increase grandparents' physical endurance as much as possible [44, 45].

It is worth noting that 60.1% of the solo grandparents viewed their physical health as “*good*” or better, suggesting most grandparents either lacked poor health conditions or were able to manage existing health conditions effectively. A similar finding was revealed in a study by Hughes et al. [21] who analyzed national data on custodial grandparents using the Health and Retirement Survey, which is administered in an older sample than BRFSS respondents. The authors suggest that the health effects of caregiving depended on the “circumstances and context” when grandchildren entered custodial care (p. S115). In particular, they found that there is a subpopulation of custodial grandparents who are susceptible to severe health risks and require health care support. However, they also revealed another group of grandparents who have significant health concerns, but who manage them well enough so that they do not interfere with their family responsibilities. Our analyses appear to align with Hughes et al.'s results, but additional research is necessary to clarify if these findings remain stable across all custodial grandparents' family arrangements, and if health severity is most apparent within certain demographic groupings, such as, age, race, gender, residential locality, or SES.

### 6.2. Mental Health

It was expected that solo grandparents would display more adverse mental health effects than single parents. However, mental health appears to be a point of commonality between solo grandparents and single parents. Our results show the prevalence of lifetime depression was worryingly high at approximately one in four in both parent groups, with minimal differences between the groups. To put this in context, in 2008 (the last year data are available) the estimated lifetime prevalence of depression for adults in the U.S. was 16.1% [46]. From this perspective, both family groups in our study have high clinical depression ratings. Presently, too little is understood about the manifestation and management of mental stress among grandparent caregivers and how these stressors may contribute to adverse mental health effects. Research that tracks the trajectory of mental health illnesses stemming from grandparent caregiving burdens needs further attention.

Solo grandparents are slightly more likely to be free of any “bad days” due to mental health concerns than single parents (61.3% versus 56.7%) each day. What actually constitutes a “bad day” or how it manifests itself in daily routines with either family group is unknown. But knowing that both single parents and solo grandparents appear to suffer high levels of mental health problems suggests this is an area of intense need for a segment of these families.

In earlier works, several authors reported that custodial grandparents experienced mental health stress as well as depressive symptomology in relationship to caregiving [47, 48]. Our findings suggest adverse mental health effects occur, but they are not necessarily a “given.” The majority of solo grandparents appear to be able to cope adequately with respect to their mental health functioning. In view of these findings, our expectation about differences in mental health attributes between solo grandparents and single parents is only partially confirmed.

### 6.3. Functional Limitations

The results assessing the functional capacity of solo grandparents are consistent with our expectations. Nearly 33% of solo grandparents experienced functional limitations due to physical or mental health conditions, as compared to a quarter of single parents. Functional limitations included limitations in usual activities, such as self-care, work, or recreation. The largest difference was between the percent of solo grandparents whose functional limitations extended beyond a week in duration (23.7%), as compared to that of single parents (11.8%). It is not clear if this finding reflects the effects of independent caregiving responsibilities, normative aging effects, or a combination of these or other factors. Previous studies have reported that grandparent caregivers experience restrictions in their daily routine functioning [49], but greater efforts are needed to assess how impaired functioning affects personal and family caregiving responsibilities among solo grandparents since family demands rest largely with them. For example, older grandchildren may take an active role in carrying out some of the physical tasks to relieve activity burdens for the grandparents. But if grandparents are raising very young grandchildren the weight of performing various functions associated with parenting (e.g., lifting/carrying infants and toddlers, bathing, preparing meals, and playing) falls to them alone. Being unable to carry them out adequately has serious implications for the health and well-being of these young grandchildren.

### 6.4. Health Behaviors

The results on the health behaviors suggest solo grandparents may have increased risks of adverse health outcomes due to poor health behaviors than single parents. The social constructs of race and poverty should be considered when interpreting these results. A substantial body of research on the health status of grandparent caregivers has focused on African American custodial grandmothers from low SES backgrounds [50]. Our data show that a disproportionate number of solo grandparents are African American, have a low SES and live in urban centres. Grandmothers of color, in particular, living in communities with limited economic opportunities, or those who experienced the cumulative effects of lifelong poverty, are at risk for poor health behaviors [51, 52]. Previous research suggests persons with low SES backgrounds are less likely to participate in physical activities, are more likely to smoke, and are at greater risk for obesity [53–56]. Our results concur with these findings, showing that two in five solo grandparents had not participated in any recreational exercise in the month preceding the survey. Furthermore, a larger percentage of solo grandparents were current smokers (28.9%), as compared to single parents (22.3%). One cannot understate the health risks these grandparents could face when considering both the lack of physical activity and smoking. Asthma, COPD, allergies, cancer, and osteoporosis are a sampling of the known harmful effects of smoking, each of which may significantly diminish one's overall physical health. Grandchildren exposed to second-hand smoke are also susceptible to poor health outcomes [53, 54]. Our findings showed nearly 16% of the grandchildren had a reported diagnosis of asthma, putting them at high risk for serious respiratory distress if they reside with a current smoker. This suggests the need to test intervention strategies designed to prevent or reduce health risks for grandparents and grandchildren.

### 6.5. Health Service Utilization

Our expectation that solo grandparents would have less use of health services as compared to single parents was guided by the literature, which suggests custodial grandparents experience personal and structural barriers that restrict their seeking and using health care services to their advantage [57]. However, the findings present a different perspective. Access to and use of health care services were higher among solo grandparents as compared to single parents. Nearly all of the grandparents reported having some form of medical insurance coverage. A large number probably received Medicare, since over 60% of grandparents were aged 60 years or over, and another portion probably received Medicaid, since their low SES background would make them eligible for public benefits. Approximately 80% of the solo grandparents reported they did not experience barriers to accessing health care services due to cost or unavailable health personnel. A similar percentage of solo grandparents had a primary health care provider and received routine physical examinations. Baker and Silverstein [26], reported that grandparents who had been raising their grandchildren for at least two years were likely to participate in health prevention activities that required the least effort (e.g., obtaining flu shots at the local drug or grocery store). However, the authors further note that the grandparents did not adhere to health prevention efforts that required more time or specific doctor appointments for more serious health issues such as pap tests or mammograms. In contrast, our data suggest that a majority of solo grandparents have done well in using health services on an annual basis. However, the high percentage of grandparents who perceive their health as “fair or poor” (39.8%) suggest a need to further explore associations between specific health care service usages, perception of health, and actual health care status because of the strong links between self-rated health and adverse health outcomes, such as morbidity and mortality [58]. Active engagement by solo grandparents in receiving health care services appears to positively benefit the grandchildren. The grandchildren had a higher rate of receiving flu shots (63.2%) and a lower prevalence of asthma (15.8%) compared to children of single parents. This finding concurs with other works suggesting when adult caregivers are engaged with the health care system for their own health conditions the children are more likely to receive age-appropriate health care services as well [59].

### 6.6. Study Limitations

The limitations of our study include the following: First, our sample consists of landline telephone users; persons who use cellular telephones as their primary communication source were not included. Second, all data are based on respondent self-report, which can be affected by recall bias. Third, the study analysis did not disaggregate the results by the respondents' demographic characteristics. Future work is warranted that explores possible effects of race, gender, SES, regional locality, and other characteristics on health and health behaviors among various family subgroups.

### 6.7. Practice and Research Implications

Comparing health characteristics of solo grandparents and single parents raises awareness about the physical and mental health status of both family groups. However, the level of poor health among at least a segment of solo grandparents who are making efforts to raise one or more grandchildren alone is especially worrisome. Efforts to increase grandparents' healthy habits, including smoking cessation, increased physical activity, and healthy eating, are required. Age, low SES, and race are attributes that should be considered in how health interventions are promoted, delivered, and received by grandparents. Health professionals working with solo grandparents should also consider how certain familial, cultural, and social attributes may increase negative consequences for physical and mental health behaviors and outcomes. Providing culturally-sensitive information about meal preparation, exercise, stress reduction, and adhering to treatment regimes are key areas where health professionals could have a positive impact.

Effective work with grandparents raising grandchildren requires an interdisciplinary approach. Physicians, nurses, and other service professionals should work collaboratively with the family to promote healthy lifestyle changes. Their efforts may not only effectively impact grandparents' health outcomes but also foster better family functioning and well-being [12, 18, 27].

Our findings provide a base for continuing research on this topic. Future studies should assess associations between various health indicators of solo grandparents in comparison with single parents or other parenting groups, moderated by factors such as race, age, gender, and SES and/or focus on neglected subgroups such as grandfather caregivers. Further study is also needed to determine what types of service meet specific needs most effectively.

## 7. Conclusion

The findings from our study highlight the vulnerability of solo grandparents, as well as single parents, to poor physical and mental health. Based on the Transactional Model of Stress and Coping [38], which framed the current study, helping solo grandparents progress beyond initial interpretation of life challenges to a point where they can begin to cope with the event (i.e., secondary appraisal) may be an area that warrants further study. Research exploring the concept of resiliency, the active adaptation to adverse life events [60], among grandparent caregivers is another important area of future research.

Our understanding about grandparents raising grandchildren continues to evolve, particularly as we learn more about how they compare with other parenting groups. The vulnerability of solo grandparents signals the need to make concerted efforts to facilitate the delivery of health services to effectively address their physical and mental health needs. Health professionals from multiple disciplines can be an important resource to reduce health problems experienced by grandparents and help increase their physical and emotional capacity to support and nurture the grandchildren in their care.

## Figures and Tables

**Table 1:** Solo grandparents and single parents: family demographic characteristics.

Grandparent's/single parent's demographic indicators	Solo grandparents %	Single parents %	*p* value
Gender			
Male	23.7%	20.4%	0.05
Female	76.3%	79.6%	
Age			
18–39	1.4%	48.3%	<0.001
40s	8.3%	35.2%	
50s	29.6%	14.5%	
60s	32.5%	1.7%	
70s	23.8%	0.2%	
80s	4.4%	0.1%	
Race			
White, non-Hispanic	51.8%	50.5%	<0.001
Black, non-Hispanic	31.2%	24.5%	
Hispanic	9.0%	18.8%	
Other races, non-Hispanic	8.0%	6.3%	
Education			
Did not graduate from high school	31.2%	14.7%	<0.001
Graduated from high school	68.8%	85.3%	
Income categories			
<$15,000	27.8%	20.2%	<0.001
$15,000–$24,999	36.4%	23.9%	
$25,000–$49,999	20.8%	24.6%	
$50,000–$74,999	7.3%	13.0%	
$75,000 or more	7.7%	18.3%	
MSA Code			
In the center city of MSA	43.5%	42%	<0.001
Outside center city of MSA	22.3%	26%	
Inside a suburban county of MSA	12.7%	13%	
In MSA with no center city	0.2%	1.1%	
Not in MSA	21.4%	15.6%	
Number of children in household			
1	61.9%	42.8%	<0.001
2	28.3%	35.4%	
3	7.3%	14.4%	
4	1.5%	5.1%	
5+	0.9%	2.3%	
Gender of grandchild			
Boy	51.0%	51.1%	0.98
Girl	49.0%	48.9%	
Age of grandchild			
<5 years	10.5%	12.9%	0.07
5–12 years	41.6%	43.8%	
13–18 years	47.8%	43.3%	

**Table 2:** Solo grandparents and single parents: family physical and mental health indicators.

Physical and mental health indicators	Solo grandparents %	Single parents %	*p* value
Arthritis			
Yes	50.5%	18.8%	<0.001
No	49.5%	81.2%	
COPD			
Yes	21.4%	4.2%	<0.001
No	78.6%	95.8%	
Ever diagnosed with diabetes (excluding borderline or gestational)			
Yes	25.6%	6.1%	<0.001
No	74.4%	93.9%	
Asthma			
Yes	22.9%	14.4%	<0.001
No	77.1%	85.6%	
Cancer (other than skin)			
Yes	13.2%	4.3%	<0.001
No	86.8%	95.7%	
Heart attack			
Yes	18.0%	1.4%	<0.001
No	82.0%	98.6%	
Stroke			
Yes	8.7%	1.6%	<0.001
No	91.3%	98.4%	
Angina or coronary heart disease			
Yes	11.2%	1.9%	<0.001
No	88.8%	98.1%	
Kidney disease			
Yes	6.9%	1.8%	<0.001
No	93.1%	98.2%	
Self-rated health			
Good or better health	60.1%	82.9%	<0.001
Fair or poor health	39.8%	16.6%	
Do not know/not sure or refused/missing	0.2%	0.4%	
Number of days when physical health was not good in past month			
No bad days	50.1%	62.2%	<0.001
1–7 bad days	18.3%	23.5%	
>1 bad week/past month	31.7%	14.4%	
Activity limitations in last month due to physical or mental illness			
No days limited	67.2%	74.6%	<0.001
1–7 days limited	9.1%	13.6%	
>1 week limited/past month	23.7%	11.8%	
Number of days when mental health was not good in past month			
No bad days	61.3%	56.7%	<0.001
1–7 bad days	14.9%	21.4%	
>1 week/past month	23.8%	21.8%	
Lifetime depressive disorder			
Yes	27.0%	23.7%	0.06
No	73.0%	76.3%	
*Health behaviors*			
Any recreational physical exercise in last month			
Yes	59.4%	75.8%	<0.001
No	40.6%	24.2%	
BMI category			
Neither overweight nor obese	32.7%	35.1%	0.10
Overweight	30.4%	32.3%	
Obese	36.9%	32.6%	
Smoking status			
Current smoker, smokes everyday	20.9%	15.8%	<0.001
Current smoker, smokes some days	8.0%	6.5%	
Former smoker	34.5%	17.8%	
Never smoked	36.5%	59.9%	
Child history of asthma			
Yes	15.8%	19.7%	0.02
No	84.2%	80.3%	
Child flu shot in past 12 months			
Yes	63.2%	50.3%	<0.001
No	36.8%	49.7%	

**Table 3:** Solo grandparents and single parents: health services utilization.

Health service utilization indicators	Solo grandparents %	Single parents %	*p* value
Have any health care insurance coverage			
Yes	91.6%	81.1%	0.00
No	8.4%	18.9%	
Have one or more personal doctors/health care providers			
Yes	89.7%	81.6%	0.00
No	10.3%	18.4%	
Could not see doctor due to cost			
Yes	18.6%	22.4%	0.02
No	81.4%	77.6%	
Length of time since last routine check-up			
<12 months	81.9%	68.4%	0.00
1-2 years	6.3%	15.2%	
3–5 years	6.2%	9.3%	
5+ years	5.6%	7.1%	
